# The feasibility of mindfulness‐based relapse prevention for adults with substance use disorders (illicit drugs) in a Chinese population: A pilot randomized controlled trial

**DOI:** 10.1111/add.70441

**Published:** 2026-04-30

**Authors:** Ka Tsun Ting, Ivan Chun Him Leung, Dicken Cheong Chun Chan, Ken On Tai Yu, Wai Kwong Tang, Fu Chan, Alan Ka Lam Tang, Helen Shuk Wah Ma, Benjamin Hon Kei Yip, Daisy Dexing Zhang, Sarah Bowen, Samuel Yeung Shan Wong

**Affiliations:** ^1^ JC School of Public Health and Primary Care The Chinese University of Hong Kong Hong Kong China; ^2^ Thomas Jing Centre for Mindfulness Research and Training The Chinese University of Hong Kong Hong Kong China; ^3^ Department of Psychiatry, Faculty of Medicine The Chinese University of Hong Kong Hong Kong China; ^4^ Department of Psychiatry North District Hospital, Hospital Authority Hong Kong China; ^5^ Department of Psychiatry Shatin Hospital, Hospital Authority Hong Kong China; ^6^ Hong Kong Centre for Mindfulness Hong Kong China; ^7^ School of Nursing, Faculty of Health and Social Sciences The Hong Kong Polytechnic University Hong Kong China; ^8^ Department of Psychology Pacific University Portland Oregon USA

**Keywords:** Chinese, illicit drug, MBRP, mindfulness, mindfulness‐based relapse prevention, substance use disorder

## Abstract

**Background and aims:**

Mindfulness‐based relapse prevention (MBRP) has been shown to be beneficial to individuals with substance use disorder (SUD) in the West. The current pilot study aimed at testing the feasibility of MBRP in a Chinese population.

**Design:**

This pilot study adopted a design of randomized controlled trial comparing MBRP with treatment‐as‐usual group (TAU).

**Setting:**

Participants were recruited from residential detox centers, community addiction counseling centers and substance abuse clinics specialized addiction treatment clinics in Hong Kong.

**Participants:**

A total of 81 adults (85.2% male) with SUD (illicit drugs only) were recruited.

**Interventions:**

The intervention group participants (*n* = 41) attended a 1‐hour orientation session followed by 2‐hour weekly MBRP sessions for 8 weeks, delivered by a qualified MBRP teacher. The TAU group participants (*n* = 40) continued their service received from their referral agency. (After completion of all study assessments they were offered the same 8‐week MBRP course.)

**Measurements:**

Feasibility was measured by attendance, course satisfaction and retention rate. Participants' change in substance use and other related outcomes were captured by self‐reported drug use, urine drug tests and a list of psychometric scales at baseline, immediately after MBRP and 3‐ and 6‐month follow‐up.

**Findings:**

The MBRP course satisfaction was high, and the attendance (57.4%) and retention rates (63.4%–85.4%) were comparable to previous trials. No statistically significant differences were observed between the MBRP and TAU groups for any outcomes, including craving, depression, anxiety, mindfulness and health‐related quality of life; however, improvement trends were noticed in the MBRP group in self‐efficacy in managing high‐risk situations at post intervention, as well as in addiction severity and psychological flexibility at the 6‐month follow‐up.

**Conclusions:**

Mindfulness‐based relapse prevention was shown to be feasible for substance use disorder treatment in a Chinese population. In this small study there was only limited evidence of abstinence efficacy, and no evidence of a benefit on other secondary outcomes.

## INTRODUCTION

Substance use disorder (SUD) related to illicit drugs is a global public health challenge. In Hong Kong, the epidemiological study on mental disorders, conducted from 2010 to 2013, reported a 2.1% 12‐month prevalence of substance dependence related to heroin, ketamine, cannabis, 3,4‐methylenedioxymethamphetamine (MDMA), methamphetamine, cocaine, hypnotics and cough mixture [1]. At the time this study started in 2019, among 5775 officially reported drug users, the most commonly reported illicit drugs were heroin (51%), methamphetamine (23.7%), tranquilizers (15.2%) and cocaine (13.9%). Cannabis (49%) and cocaine (44%), however, showed a stronger trend among young users under the age of 21 [2]. Comparatively, United States showed a higher prevalence (9.6%) of illicit drug use disorders, driven by cannabis and opioids, while England reported 3.1%, with cannabis predominant [3, 4].

Illicit drug use has brought a significant economic burden to Hong Kong society, with an estimated tangible cost of USD$1.32 billion in 2014. This cost was attributed to productivity loss, healthcare expenditures, law enforcement and social services [5]. Illicit drug use was also associated with higher rate of mortality, attendance to emergency service and hospital admissions in Hong Kong [6]. These challenges emphasize the urgent need for effective interventions to address illicit drug‐related SUD and to alleviate the economic burden on society.

In Hong Kong, illicit drug‐related policies and services are coordinated by separate governmental departments from those addressing tobacco and alcohol use [7, 8]. The major types of drug treatment and rehabilitation services in Hong Kong included compulsory placement scheme provided by the correctional department, specialized clinics in public hospitals, voluntary residential programs and community counseling centers operated by non‐governmental organizations. These services often integrate evidence‐based psychological interventions, such as cognitive behavioral therapy (CBT) and motivational interviewing (MI) [9, 10, 11]. However, only 24% of individuals with psychotropic substance dependence in Hong Kong sought professional help [1]. A meta‐analysis indicated that CBT, when applied to Chinese population, yielded only modest, short‐term reductions in addictive behaviors [12]. Prior studies suggested traditional CBT techniques may be less effective in modifying dysfunctional beliefs among Chinese population [11, 12]. These findings highlight the need for more innovative and culturally attuned interventions to improve engagement and treatment effectiveness.

Mindfulness is defined as the awareness that arises when paying attention on purpose to the present moment in a non‐judgmental way [13]. A systematic review and meta‐analysis provided initial evidence that mindfulness training may reduce substance use, cravings and stress in individuals with SUDs [14]. A subsequent Cochrane review also found mindfulness‐based interventions (MBIs) slightly reduced substance use at post‐treatment and follow‐up, compared to other interventions, but the evidence was uncertain when MBIs compared to no intervention and in other substance‐related outcomes [15]. In Hong Kong, where approximately one million individuals (13.3% of the total population) follow Buddhism and one million follow Taoism, meditation is a familiar component of these widely practiced religions [16]. Additionally, mind and body exercises, such as Tai Chi, Qigong and yoga, are widely embraced locally. Given this cultural context, MBIs are likely to be well‐received, offering a culturally attuned approach to address cravings and dysfunctional beliefs associated with substance use.

Mindfulness‐based relapse prevention (MBRP) is among the most rigorously studied MBIs for SUD [17, 18]. Its strength includes:

1. Integration with evidence‐based relapse prevention (RP): MBRP combines mindfulness training with RP, a CBT approach with established efficacy for SUD [19, 20]. Beyond traditional RP skills, participants also learn to disengage from automatic reactions to craving, de‐center from the beliefs driving substance use, foster acceptance toward unpleasant experiences and respond wisely through cultivating moment‐by‐moment awareness [21].
2. Craving reduction: MBRP was found to be effective in reducing craving, a core diagnostic criterion of SUD [22]. A sub‐group analysis from a recent meta‐analysis reported a moderate effect size (Cohen's *d* of −0.59) for MBRP in reducing cravings [23].
3. Adaptability to comorbid conditions: MBIs have demonstrated efficacy in addressing comorbid conditions, such as depressive symptoms and stress, which are frequently associated with SUD [24, 25]. In Hong Kong, where illicit drug use is frequently motivated by the desire to avoid discomfort and alleviate negative emotions [2], MBRP's focus on cultivating non‐judgmental awareness offer a promising approach in addressing these co‐occurring conditions.
4. Cultural and practical advantages of group format: MBRP is designed for delivery in a group format, making it a more cost‐effective compared to individual therapy. Group‐based format also aligns with Hong Kong's collectivist culture, where social interconnectedness is valued and fosters peer support, which might potentially enhance participant engagement and treatment adherence.To date, only one meta‐analysis evaluated MBRP, reporting a small effect for reducing withdrawal symptoms, craving and negative consequences of substance use, although the confidence in these findings was limited because of methodological limitation [26]. More rigorously designed studies are needed to confirm MBRP's effectiveness. Additionally, most MBRP trials were conducted in the West. At the time of writing this study protocol in 2018, there were six RCTs evaluating MBRP in non‐Western populations, but five of them were from Iran [27, 28, 29, 30, 31]. There was only one study from Taiwan, and it had a small sample size of 24 participants [32]. It was also conducted in an incarcerated setting and only had pre‐post comparison. Therefore, it was unknown if the benefit of MBRP could generalize into the Chinese population. This pilot study aimed to replicate the first MBRP RCT [17], while evaluating the feasibility of implementing MBRP within the Chinese population in a naturalistic setting.

## OBJECTIVES

Our objectives were (1) to evaluate the feasibility and acceptability of MBRP for adults with SUD (illicit drugs) in Hong Kong; and (2) to explore the potential effectiveness of MBRP, compared to a treatment‐as‐usual group (TAU), in changes in substance use, craving, mood and anxiety symptoms, self‐efficacy, psychological flexibility, mindfulness and health‐related quality of life.

## METHODS

### Design

This feasibility study adopted an open‐label, two‐arm RCT design to compare MBRP with the TAU group. The intervention group was provided with 2‐hour weekly MBRP sessions for 8 weeks, delivered by a qualified MBRP teacher, while TAU group continued to receive services from the corresponding referral agencies. Participants in the MBRP group also received the same usual care services as those in the TAU group. Follow‐ups were conducted immediately after MBRP and 3‐ and 6‐month follow‐up. After completion of all study assessments, the same 8‐week MBRP course was offered to participants in the TAU group.

### Study population

Eligibility was determined by the following criteria. Inclusion criteria included: (1) between 18 and 70 years old; (2) fluent in Cantonese; (3) had ever met the Structured Clinical Interview for Diagnostic and Statistical Manual of Mental Disorders (DSM)‐IV/DSM‐5 criteria for SUD with their primary substance being illicit drugs (heroin, cocaine, methamphetamine, cannabis, tranquilizer, ketamine, cough medicine and MDMA as defined by the Narcotics Division of Security Bureau in Hong Kong); and (4) had completed outpatient or inpatient SUD treatment in the previous 6‐month or was currently participating in community‐based rehabilitation service. Exclusion criteria included (1) unable to provide a valid consent; (2) actively psychotic; (3) had an imminent suicide risk; or (4) had dementia.

### Procedures

#### Recruitment and consent

To assist with recruitment, the research team engaged with various parties that offer community and residential detox services. A small group of participants were recruited from another men's health study. A trained research assistant screened willing referrals from these sources to ensure their eligibility before they were invited to join and provide informed consent.

#### Randomization, concealment and blinding

The therapist and participants were not blinded in this open‐label trial. Participant recruitment took place from October 2019 to May 2023, spanning the coronavirus disease 2019 (COVID‐19) pandemic period in Hong Kong, which began in January 2020 and lasted through early 2023, with multiple waves of infection and strict public health measures. Because of the constraints of the policies of the collaborating parties, COVID‐19 restriction policy and confidentiality, it was not possible to combine eligible participants from different referral sources into a single group. Instead, each referral source had its own randomization sequence, which was generated by an independent statistician. The staff from each referral source were kept blinded about the group allocation sequence. Within each referral source, simple randomization was used. The participants were notified of their group assignment result through the staff of their corresponding referral source.

##### Treatment and follow‐up timeline

After collection of baseline data and randomization, MBRP group started the 8‐week intervention program (see the following section). At 8 weeks (immediately post‐intervention), 3 months and 6 months, outcome data were collected from both groups by the research assistant.

#### Intervention and control group

##### MBRP

The MBRP group in this study followed the traditional 8‐week manualized protocol with 2‐hour weekly sessions for 8 weeks plus a 1‐hour orientation session before the group began [33, 34]. In the orientation session, participants received a brief psycho‐education on the benefit of mindfulness practice, and they were offered a sample 5‐ to 10‐minute mindfulness of body and breath practice. The importance of home practice was emphasized.

Each MBRP session followed a similar structure. Sessions typically started with a mindfulness practice, followed by home practice review, then a cognitive exercise related to the theme of the session or an introduction of a new mindfulness practice and ending with suggesting home practice followed by a brief closing mindfulness practice.

Participants had the chance to share their experiences in the class. The MBRP teacher would adopt an inquiry‐based approach to facilitate participants' learning. Handouts and audio recordings were given to the participants to facilitate their home practice. A detailed description of the intentions and activities for each session is included in Appendix 1.

MBRP group was delivered in Cantonese (a dialect of spoken Chinese). The handouts and audio recordings were originally in English. Before this study, there were no available Chinese translated materials. The MBRP materials were first translated by a research assistant, who was fluent in with both English and Chinese, and fine‐tuned by the MBRP teacher to align the wordings with the spirit of mindfulness. Cultural adaptation was made during the translation process. For example, ‘SOBER space’ in the original MBRP was changed to ‘3‐step breathing space’, as most of the participants did not understand the meaning of the English word ‘SOBER’. The adapted protocol had a trial run to collect feedback before the start of this study.

The MBRP group was delivered by a clinical psychologist, who was a qualified MBRP teacher, with experience in teaching MBIs for more than 10 years. The MBRP teacher also had a training background in MI and CBT for addiction. The MBRP teacher received ongoing supervision from an experienced MBRP trainer and supervisor when delivering the group.

The format of MBRP in this trial was originally designed to be face‐to‐face. Most of the groups were conducted face‐to‐face, but some sessions changed to video conferencing via Zoom because of social distancing measures during the COVID‐19 pandemic period. Of the 41 participants who received MBRP, 19 attended sessions in person, 16 participated via Zoom and 6 received the intervention in a hybrid format.

##### Intervention fidelity

All MBRP sessions were audio recorded. One fourth of the MBRP sessions were selected by an external statistician for fidelity assessment. The assessment was conducted by an external mindfulness teacher, with a clinical psychologist background, based on an adapted version of Mindfulness‐based Relapse Prevention Adherence and Competence Scale [35].

##### TAU

Participants in the TAU group continued their service received from their referral agency. The services included an exercise group, individual counseling or pharmacological intervention based on their needs. Participants were offered the same 8‐week MBRP course after they completed all study assessments. Participants could decide if they wanted to join the MBRP group a voluntary basis.

#### Measures

All data were collected by two trained research assistants. All questionnaires were administered in written Chinese.

##### Demographic information

Demographic information, such as age, gender, education level, marital status and personal income were collected at the baseline assessment.

##### Primary outcome—Feasibility and acceptability

Feasibility was measured by the recruitment and retention rate. The trial that this study aimed to replicate had a retention rate of 61% at post‐intervention and 73% at 4‐month follow‐up [17]. Therefore, it was considered feasible if the retention rate was not lower than 61%. Acceptability was measured by course attendance, course satisfaction and adverse events. A questionnaire was designed to measure course satisfaction at post‐intervention in which participants rated, using a 10‐point Likert scale, their overall satisfaction, perceived importance of the program and how likely they would continue mindfulness practice after the course. Adverse events were monitored through participant self‐reports.

##### Secondary outcomes

Substance use was measured at all time points by participants' self‐reported illicit drug use in the past 7 days. Self‐reported abstinence from illicit drug use was validated by urine test. For analysis, substance use was coded as non‐abstinence (any illicit drug use), to reflect reductions in use as a positive treatment outcome. The severity of the substance use in the past 30 days was measured by Addiction Severity Index‐Lite version (ASI) [36]. ASI is a semi‐structured interview and covers different areas, such as medical, employment, drug and alcohol use and legal issues. In this study, only the drug use section was used [37]. ASI had been translated in Chinese and applied in local study [38]. A higher composite score indicated a higher level of addiction severity.

Craving was measured by modified version of Penn Alcohol Craving Scale (PCS) [39]. The wording of the scale was adapted to measure illicit drug use. This 5‐item self‐report scale measured the frequency, intensity and duration of the craving experience. Studies have shown that a modified version of PCS for substance use shows good short‐term test–retest reliability, long‐term sensitivity and factor structures [40]. The internal consistency of PCS was 0.92 in this study.

Depression and anxiety were measured by the 9‐item Patient Health Questionnaire (PHQ‐9) and 7‐item Generalized Anxiety Disorder (GAD‐7) [41, 42]. Both scales demonstrated good reliability and validity. The internal consistency of PHQ‐9 and GAD‐7 in this study was 0.91 and 0.94, respectively.

Participants' self‐efficacy in managing high‐risk situations was measured by Drug Avoidance Self‐Efficacy Scale (DASES) [43]. It has 16 items in total, with one high risk situation mentioned in each item. Participants were asked to rate how likely they would be able resist the temptation, using a 7‐point scale. Higher score in DASES represented a higher self‐efficacy. The internal consistency was acceptable (i.e. 0.85) in this study.

Level of psychological flexibility and mindfulness were captured by The Acceptance and Action Questionnaire‐second edition (AAQ‐II) and Five Facet Mindfulness Questionnaire (FFMQ) respectively [44, 45]. The internal consistency of AAQ‐II and FFMQ in this study was 0.91 and 0.69, respectively.

Health‐related quality of life is measured by EuroQol 5‐dimensions instrument with five‐level scale (EQ‐5D‐5L) Hong Kong (HK) version [46]. EQ‐5D‐5L has been culturally adopted and widely used in local setting to measure the health‐related quality of life [46, 47, 48].

Participants in the intervention group were also invited to provide qualitative feedback on their overall experience of the course after they completed MBRP.

### Statistical analyses

#### Primary outcome measures

Descriptive statistics were used to summarize the primary outcomes for each arm. Counts and percentages were reported for retention and adverse events, while means and SDs were calculated for course attendance and evaluation scores. Retention rates between the two groups were compared using χ^2^ tests.

#### Secondary outcome measures

With the exception of substance use, addiction severity index and health‐related quality of life, internal consistency of secondary measures assessed at baseline was evaluated using Cronbach's α. Generalized estimating equation (GEE) was used to analyze abstinence status (categorical) across multiple time points, while linear mixed models (LMM) were used for the analysis for all other secondary outcomes. Both GEE and LMM provided robust handling of missing data under the assumption that the data were missing at random (MAR). Given the observed non‐linear trends, time was treated as a categorical variable. Intervention group, time and their interaction were specified as fixed effects. A random intercept for each subject was included to account for individual variability. To model within‐subject correlations over time, an autoregressive (AR1) covariance structure was applied. Baseline characteristics found to be predictive of the outcome (*P* < 0.1) were included as covariates in the adjusted comparison analysis to account for potential confounding. Specifically, age was associated with substance use, PCS, PHQ‐9, GAD‐7 and AAQ‐II, while gender was predictive of PHQ‐9, GAD‐7, AAQ‐II, FFMQ, EQ‐5D‐5L and EQ‐5D‐5L health score. Education level showed associations with PCS, GAD‐7 and FFMQ, whereas marital status was related to PHQ‐9, GAD‐7, ASI, AAQ‐II, FFMQ and EQ‐5D‐5L. Employment status was predictive of FFMQ and EQ‐5D‐5L health score. These variables were incorporated into the adjusted analyses for each respective outcome. All intervention effects presented using GEE and LMM models were based on adjusted analyses. Between‐group effect sizes (Cohen's *d*) were calculated using the mean differences from baseline and the SDs of the two groups at each time point. A two‐sided *P*‐value of <0.05 was considered statistically significant. SPSS version 26.0 (SPSS) was performed for all analyses.

### Sample size calculation

The trial was originally designed to evaluate the effectiveness of MBRP, with self‐reported drug use and urine drug test results as the primary outcomes. In Bowen *et al*. [49], participants in the MBRP and cognitive‐behavioural relapse prevention groups showed a 54% decreased risk of relapse to drug use at 6‐month follow‐up compared to those receiving TAU, indicating a medium to large effect size. Conservatively, medium effect of 0.5 was applied for sample size estimation. A fully powered trial with 80% power and a two‐sided α of 0.05 would require 64 participants per group. However, because of recruitment challenges during the COVID‐19 pandemic, the study was revised to a pilot design, with feasibility and acceptability as the primary outcomes. It has been recommended to have at least 12 participants for each group in a pilot study [50]. The average drop‐out rate of psycho‐social treatment for individuals with SUD was approximately 30%, and it was even higher for studies targeting illicit drug use [51]. Considering high drop‐out rate in this study population and the relatively long follow‐up of the study, the research panel increased the sample size to 40 participants in each group (i.e. 80 in total) to prevent the loss of statistical power and potential bias and to provide preliminary effect size estimates to inform future trial planning.

### Ethical consideration

This study followed the Code of Ethics of the Declaration of Helsinki. Ethics approval was obtained from the Joint Chinese University of Hong Kong‐New Territories East Cluster Clinical Research Ethics Committee (CREC ref no.: 2019.230‐T) before the trial. This trial was registered at ClinicalTrials.gov (Trial Registration Number: NCT04034732; date of registration: 25 July 2019) and the Chinese Clinical Trial Register (Trial Registration Number: ChiCTR1900026247; date of registration: 28 September 2019). Written consent was obtained from all participants before enrolment, and confidentiality of participant data was strictly maintained throughout the study.

## RESULTS

### Recruitment and participant flow

**FIGURE 1:**
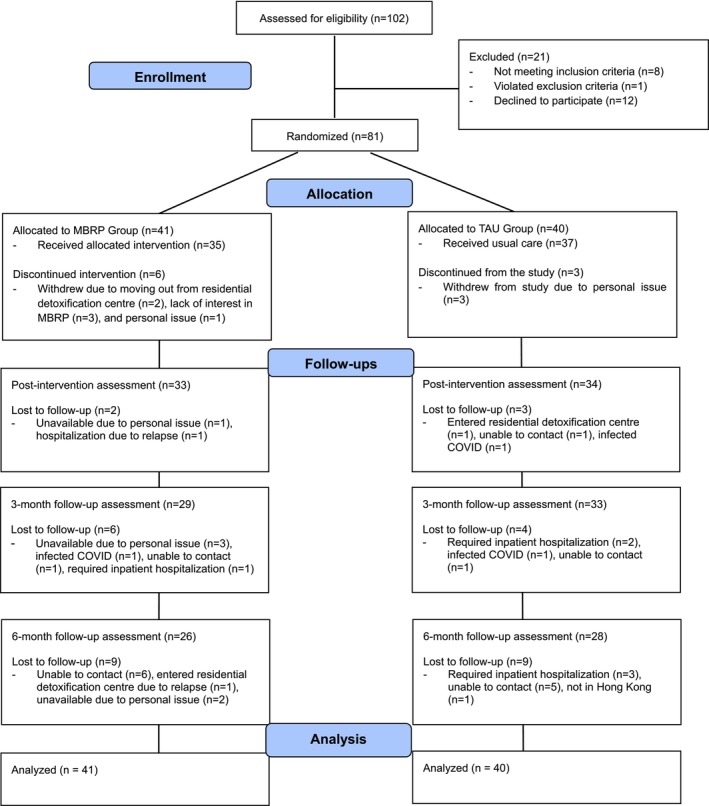
The CONSORT (Consolidated Standards of Reporting Trials) flow diagram of the study.

Among 102 participants screened, 81 (79.4% recruitment rate) were eligible and successfully recruited to the study. The study flow is shown in Figure 1 according to the Consolidated Standards of Reporting Trials Statement (CONSORT). All 81 participants were included in the analysis according to the intention‐to‐treat principle.

### Baseline data

Participant demographic information is shown in Table 1. Most of the participants were male, single and had an education level at or above secondary school. There was a noticeable difference in the baseline non‐abstinence rate between two groups (MBRP: 61% versus TAU: 30%). The potential reasons behind the significant difference (such as COVID‐19 social distancing measures and difference in referral sources) were investigated, but they could not explain the above difference.

**TABLE 1:** Baseline information of participants.

Items	MBRP (*n* = 41)	TAU (*n* = 40)
Age at enrolment, years	40.1 (10.4)	34.1 (7.1)
Male (%)	85.4	90
Education (%)		
No formal education	0	0
Primary school	2.4	5
Secondary school	68.3	65
Preparatory school or above	29.3	30
Employment (%)^a^		
Full‐time	38.5	47.5
Part‐time	23.1	17.5
Unemployed	35.9	32.5
Housewife/retired	2.6	2.5
Marriage (%)^b^		
Singled/divorced/widow	75.0	92.3
Married/cohabitation	25.0	7.7
Income (%)^c^		
No income/ <$9999	45.9	25.9
$10 000–19 999	16.2	14.8
$20 000–29 999	10.8	18.5
$30 000–$39 999	5.4	14.8
Above $40 000	21.6	25.9
Type of illicit drug use in the past 7 days (*n*)^d^		
Methamphetamine	5	5
Cannabis	2	2
Cough medicine	2	2
Ketamine	3	0
Tranquillizer	2	1
Cocaine	2	2
Heroin	1	0
MDMA	1	0
Others (e.g. paint thinner, benzodiazepine, phencyclidine)	11	5
Non‐abstinence status (%)	61.0	30.0
Penn Craving Scale	9.41 (9.23)	6.55 (7.07)
9‐item Patient Health Questionnaire	9.51 (7.96)	7.21 (5.69)
7‐item Generalized Anxiety Disorder	7.00 (6.70)	5.75 (6.00)
Addiction Severity Index‐Lite version	0.044 (0.065)	0.016 (0.032)
Drug Avoidance Self‐Efficacy Scale	73.85 (22.46)	79.58 (15.43)
Acceptance and Action Questionnaire‐second edition	24.51 (11.88)	22.73 (10.44)
Five Facet Mindfulness Questionnaire	60.93 (9.68)	59.33 (9.81)
Health‐related quality of life (EQ‐5D‐5L_Health Index)	0.80 (0.23)	0.87 (0.13)
Health‐related quality of life (EQ‐5D‐5L_Overall Health)	69.05 (19.62)	72.10 (18.00)

*Note*: Mean (SD) for continuous variables.

Abbreviations: EQ‐5D‐5L, EuroQol 5‐dimensions instrument with five‐level scale; MBRP, mindfulness‐based relapse prevention; MDMA, 3,4‐methylenedioxymethamphetamine; TAU, treatment‐as‐usual.

^a^ Unanswered: 2.

^b^ Unanswered: 2.

^c^ Unanswered: 17.

^d^ Some participants reported taking more than one kind of drug.

### Primary outcomes—Feasibility and acceptability

Participants attended an average of 4.59 (SD = 2.63) of 8 sessions in the MBRP group and 66% of them attended at least half of the sessions. Six participants (14.6%) in the MBRP group dropped out from the study because of discharge from residential detoxification center (2 participants), disinterest in MBRP group (3 participants) and personal issues (1 participant). Three participants (7.5%) in the TAU group dropped out from the study because of personal issues, including scheduling conflicts with other classes and work obligations. The total retention rate was 88.9%, which was above the 61% cut‐off, and was comparable to the original trial [17].

Regarding the follow‐up assessments, the retention rates at post‐intervention, 3‐ and 6‐month follow‐up were 80.5%, 70.7% and 63.4%, respectively for the MBRP group. The retention rates were slightly higher for the TAU group with 85%, 82.5% and 70% for post‐intervention, 3‐ and 6‐month follow‐up. However, none of the between‐group differences at any follow‐up were statistically significant. The main reason for loss to follow up in both groups was not being able to contact the participant. The summary was shown in Table 2.

**TABLE 2:** Attendance and retention rates of the MBRP program.

Items	Mean	SD	Rate (%)
No. of sessions attended (out of 8)	4.59	2.63	
Average attendance rate			57.4
Retention rates			
During 8‐week MBRP course			85.4
At post‐intervention			80.5
At 3‐month follow‐up			70.7
At 6‐month follow‐up			63.4

Abbreviation: MBRP, mindfulness‐based relapse prevention.

There were 16 participants who submitted the evaluation form after MBRP course. They reported on a 10‐point scale that MBRP was important to them [mean (M) = 8.31; SD = 1.35] and helpful (M = 8.19; SD = 1.87). They were highly satisfied (M = 8.31, SD = 1.66) and would very likely recommend this course to their friends or individuals affected by substance use problems (M = 7.75, SD = 1.81). They also reported a high chance of continuing mindfulness practice after the course (M = 7.31, SD = 2.44). The summary was shown in Table 3. Follow‐up assessment outcomes showed no noticeable differences between participants who completed the course evaluation and those who did not.

**TABLE 3:** Participants' Ratings of the MBRP program.

Items	Mean	SD
How important is this program to you?	8.31	1.35
How likely are you going to continue engaging in mindfulness practice after this course?	7.31	2.44
Would you recommend this course to your friends or people affected with addiction problems?	7.75	1.81
Overall, how helpful do you think this course is for you	8.19	1.87
Overall, how satisfied are you with this course?	8.31	1.66

### Adverse events

Although four participants reported relapse to substance use, which resulted in hospitalization or admission to detoxification center, these events were not directly related to the participation in MBRP. The adverse event in the intervention group involved a participant who attended only one MBRP session. This participant had a positive substance status at baseline and was hospitalized because of drug relapse 2 months after the 8‐week course. According to the social worker from the referral source, the hospitalization was attributed to the participant's pre‐existing condition rather than the MBRP intervention. In the control group, all adverse events were reported during the waitlist period, before participants began the MBRP program. All three participants in the control group experienced drug relapse that required hospitalization. They were subsequently discharged and admitted to a detoxification center for follow‐up treatment.

### Secondary outcomes

**FIGURE 2:**
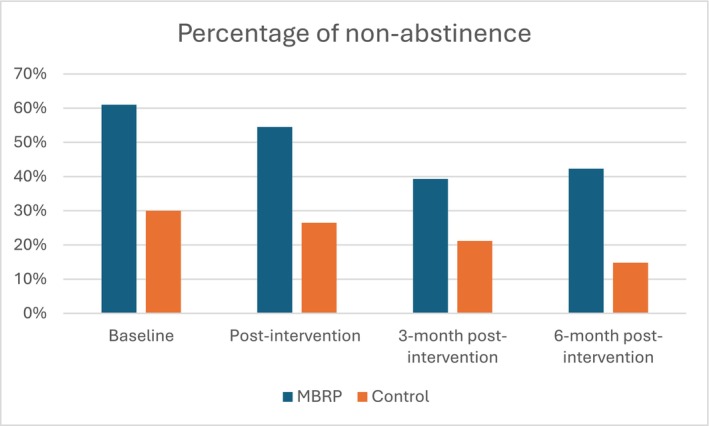
Percentage of non‐abstinence from baseline in mindfulness‐based relapse prevention (MBRP) and treatment‐as‐usual (TAU) groups.

The result of non‐abstinence status is presented in Table 4 and Figure 2. No significant between‐group difference was observed at any follow‐up time points. To explore differences by delivery format, non‐abstinence rates were compared between the hybrid/online and face‐to‐face groups. For the hybrid/online group, the rates at baseline, post‐intervention, 3‐month and 6‐month follow‐up were 50.0%, 55.6%, 37.5% and 35.7%, respectively. For the face‐to‐face group, the corresponding rates were 73.7%, 53.3%, 41.7% and 50.0%. No statistically significant differences were found between the two formats across all time points.

**TABLE 4:** Non‐abstinence status analysed by generalised estimating equations.

Non‐abstinence status	Percentage if count (non‐abstinence)		B‐coefficient (group × time)
	MBRP	TAU	
Baseline	61.0%	30.0%	
Post‐intervention	54.5%	26.5%	0.048
3‐month follow‐up	39.3%	21.2%	−0.088
6‐month follow‐up	42.3%	14.8%	0.326

Abbreviations: MBRP, mindfulness‐based relapse prevention; TAU, treatment‐as‐usual.

**FIGURE 3:**
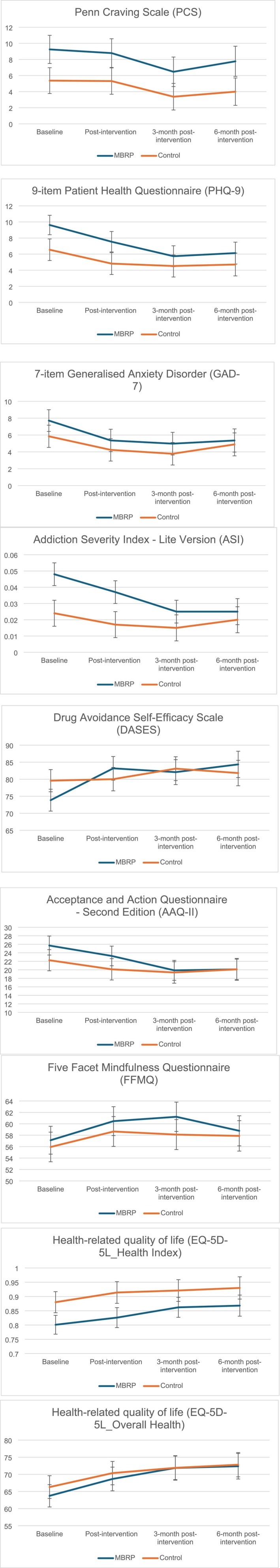
Mean change of scores and index from baseline in both mindfulness‐based relapse prevention (MBRP) and treatment‐as‐usual (TAU) groups. Note: Data are presented as estimated mean and standard error from linear mixed models.

A noticeable time‐by‐treatment interaction effect was observed for self‐efficacy in avoiding drug use at post‐intervention and in severity of substance use at 6‐month follow‐up (see Table 5 and Figure 3 for details). For self‐efficacy, the mean score in the MBRP group showed a greater increase compared to the TAU group at post‐intervention (*P*‐value = 0.056; effect size = 0.51), indicating a trend toward improved outcome. Participants in the MBRP group reported greater improvement from baseline in their self‐efficacy of handling high risk immediately after the course, but this between‐group difference did not maintain at later time points. For severity of substance use, the mean score of the MBRP group showed a greater reduction at 6‐month follow‐up compared to baseline, relative to the TAU group (*P*‐value = 0.054; effect size = 0.37). Noticeable improvements in the severity were observed in both groups across follow up period. However, a greater decreasing trend was observed in the MBRP group at the 6‐month follow‐up compared to the TAU group. Although these differences did not reach statistical significance, the observed effect sizes suggest potential clinical relevance and highlight the need for larger, adequately powered trials to confirm these findings.

**TABLE 5:** Secondary outcomes using linear mixed models.

	Mean (SD)		Estimate (group × time)	Effect size (95% CI)
	MBRP	TAU		
Penn Craving Scale				
Baseline	9.41 (9.23)	6.55 (7.07)		
Post‐intervention	9.36 (9.78)	6.50 (7.56)	−0.404	−0.04 (−0.52 to 0.44)
3 months	6.28 (7.06)	4.79 (5.53)	−0.779	0.01 (−0.49 to 0.51)
6 months	7.73 (8.03)	5.18 (6.15)	−0.108	−0.02 (−0.55 to 0.52)
9‐item Patient Health Questionnaire				
Baseline	9.51 (7.96)	7.21 (5.69)		
Post‐intervention	8 (6.38)	5.50 (5.24)	−0.355	−0.14 (−0.62 to 0.34)
3‐months	5.45 (4.71)	5.27 (4.52)	−1.858	−0.26 (−0.76 to 0.24)
6 months	5.81 (5.50)	5.50 (4.68)	−1.652	−0.33 (−0.87 to 0.21)
7‐item Generalized Anxiety Disorder				
Baseline	7.00 (6.70)	5.75 (6.00)		
Post‐intervention	5.06 (4.95)	4.24 (3.79)	−0.748	−0.19 (−0.67 to 0.30)
3 months	4.07 (3.63)	3.82 (4.13)	−0.666	−0.02 (−0.51 to 0.48)
6 months	4.46 (4.62)	5.04 (4.43)	−1.403	−0.29 (−0.82 to 0.25)
Addiction Severity Index‐Lite version				
Baseline	0.044 (0.065)	0.016 (0.032)		
Post‐intervention	0.031 (0.040)	0.009 (0.019)	−0.004	−0.07 (−0.55 to 0.41)
3 months	0.014 (0.027)	0.007 (0.013)	−0.013	−0.15 (−0.65 to 0.35)
6 months	0.016 (0.032)	0.011 (0.027)	−0.019	−0.37 (−0.91 to 0.17)
Drug Avoidance Self‐Efficacy Scale				
Baseline	73.85 (22.46)	79.58 (15.43)		
Post‐intervention	82.67 (24.52)	80.12 (18.23)	8.898	0.51 (0.02 to 0.99)
3 months	83.79 (23.24)	83.09 (20.58)	4.694	0.19 (−0.31 to 0.69)
6 months	83.77 (21.49)	83.61 (17.11)	8.267	0.38 (−0.16 to 0.92)
Acceptance and Action Questionnaire‐second edition				
Baseline	24.51 (11.88)	22.73 (10.44)		
Post‐intervention	21.94 (10.06)	20.94 (9.56)	−0.308	−0.01 (−0.49 to 0.47)
3 months	18.10 (9.11)	20.09 (10.29)	−2.948	−0.12 (−0.62 to 0.38)
6 months	18.54 (7.92)	21.32 (9.55)	−3.454	−0.46 (−1.00 to 0.09)
Five Facet Mindfulness Questionnaire				
Baseline	60.93 (9.68)	59.33 (9.81)		
Post‐intervention	64.00 (9.02)	61.47 (8.90)	0.634	0.13 (−0.35 to 0.61)
3 months	64.83 (10.48)	60.91 (9.48)	1.934	0.19 (−0.31 to 0.69)
6 months	61.61 (11.04)	59.75 (8.67)	−0.296	0.03 (−0.50 to 0.57)
Health‐related quality of life (EQ‐5D‐5L_Health Index)				
Baseline	0.80 (0.23)	0.87 (0.13)		
Post‐intervention	0.81 (0.25)	0.91 (0.09)	−0.009	0.04 (−0.44 to 0.52)
3 months	0.86 (0.19)	0.91 (0.09)	0.020	0.11 (−0.39 to 0.61)
6 months	0.86 (0.13)	0.92 (0.09)	0.017	0.15 (−0.38 to 0.69)
Health‐related quality of life (EQ‐5D‐5L_Overall Health)				
Baseline	69.05 (19.62)	72.10 (18.00)		
Post‐intervention	74.33 (13.47)	75.82 (13.12)	0.838	0.11 (−0.37 to 0.59)
3 months	78.59 (12.33)	77.55 (13.41)	2.518	0.18 (−0.33 to 0.68)
6 months	79.02 (12.22)	78.04 (12.52)	2.107	0.19 (−0.35 to 0.72)

Abbreviations: EQ‐5D‐5L, EuroQol 5‐dimensions instrument with five‐level scale; MBRP, mindfulness‐based relapse prevention; TAU, treatment‐as‐usual.

For other secondary outcomes, no significant between‐group differences were found. An effect size of 0.46 was observed in psychological flexibility measured by AAQ‐II at the 6‐month follow‐up, which suggested a possible advantage for participants in the MBRP group. This indicated that they may have experienced greater psychological flexibility compared to those in the TAU group.

## DISCUSSION

### Main findings

This study demonstrated MBRP was acceptable and feasible in the Chinese population. MBRP average attendance in this study (4.59 sessions) was comparable to other studies (4.2–6.45 sessions) [17, 52, 53]. The recruitment rate was satisfactory. Although the retention rate of the MBRP group was slightly lower than the TAU group, it was comparable to, or even better than, some of the previous MBRP studies [17, 49, 52, 53]. The majority of participants were male (87.3%), which was comparable to the government data that 80% of the reported illicit drug users in Hong Kong were male [2].

The course satisfaction was high, and participants rated MBRP helpful and important. They also gave a high scoring regarding chance of continuing mindful practice after the course and willingness to recommend MBRP to individuals who had a substance use problem. This suggests MBRP may be well‐suited to the local context.

There was no significant difference in the non‐abstinence rate between the two groups at any follow‐up time points. Interestingly, however, the MBRP group showed a trend of greater drop in addiction severity compared to TAU group at 6‐month follow‐up, suggesting that participants might benefit from MBRP in terms of addiction severity in the long term. This is consistent with some of the previous findings. For example, one study showed that participants in the MBRP group had less frequent drug use and less heavy drinking at the 12‐month follow‐up compared to both active control and TAU group, which was not seen in earlier time points [49]. In future studies, it might be interesting to investigate the exact mechanism behind the long‐term benefit of mindfulness training.

No significant differences between the two groups were found on measures of craving, depression, anxiety, mindfulness or health‐related quality of life, with the exception of a trend toward greater psychological flexibility in the MBRP group at the 6‐month follow‐up. Although this study did not replicate the benefits of MBRP group in reducing craving or enhancing acting with awareness, the observed trend toward improved self‐efficacy and psychological flexibility suggests that future investigation with a larger sample size is warranted to explore this potential effect [17].

Although no adverse events were reported directly related to attending the MBRP group or practicing mindfulness, participants reported challenges when confronting their unpleasant emotions, especially when they practiced at home, in the evaluation interview after the class. In future, group practice sessions could be arranged between each class to facilitate their practice and provide guidance to working with difficult emotions. Learning to be with unpleasant internal experience, without suppressing or avoiding it, can be very valuable as experiential avoidance often plays an essential role in addiction [54, 55]. It will also be interesting to assess the role of trauma history or post‐traumatic stress disorder in these challenges. Participants also gave feedback about the preference for face‐to‐face groups versus video conferencing format. Although this study was underpowered to detect statistically significant differences, future research could explore this further by comparing different modes of delivery.

### Study limitations

Alongside the study's findings, there are limitations to be considered. First, this study did not have an active control group. It is, therefore, uncertain whether the improvement can be attributed to generic factors, such as group support, or factors specific to the MBRP group.

The significant baseline difference in the non‐abstinence status between two groups could not be explained by available data, nevertheless, this observed variation may reflect differences in participants' recovery needs or stages. This difference might affect participants' response to MBRP group. The social distancing policy also forced a change in the delivery mode from face‐to‐face to online conferencing for some of the sessions. This change in format might have affected participants' learning, especially because tele‐health formats were still relatively new at that time. Data on other clinical psychiatric diagnoses or characteristics were not collected, which limits the ability to characterize the clinical profile of the participants.

Although the sample size of this study was not small compared to other MBRP studies, it limited the ability to detect the efficacy of the intervention. In future full trial, a fully powered sample, in addition to an active control condition, might better determine the effects of MBRP in the Chinese population.

Although participants reported high satisfaction with MBRP, the results should be interpreted with caution because of the low return rate of the evaluation form, with only approximately 40% of participants in the intervention group completing it. Although there were no noticeable differences in the outcomes between participants who completed the course evaluation and those who did not, it would be more comprehensive to collect feedback from those who did not return the evaluation form. For example, conducting an exit interview via phone or video conferencing might provide valuable insight.

## CONCLUSION

This pilot study on MBRP in the Chinese population has its strength in its relatively large sample size and long follow‐up period. MBRP was shown to be both feasible and acceptable within the Hong Kong context. Although no significant improvements were observed across measures, participants in MBRP group showed trends toward enhanced self‐efficacy in managing high‐risk situations, reduction in addiction severity and improved psychological flexibility. Future studies with a larger sample size and an active control group might provide more solid conclusions on the efficacy of MBRP in the Chinese population.

## AUTHOR CONTRIBUTIONS

**Ka Tsun Ting:** Conceptualization (equal); methodology (equal); project administration (lead); writing—original draft (lead); writing—review and editing (lead). **Ivan Chun Him Leung:** Data curation (equal); formal analysis (supporting); investigation (lead). **Dicken Cheong Chun Chan:** Data curation (equal); formal analysis (lead); visualization (lead); writing—review and editing (equal). **Ken On Tai Yu:** Visualization (supporting); writing—review and editing (supporting). **Wai Kwong Tang:** Writing—review and editing (equal). **Fu Chan:** Writing—review and editing (equal). **Alan Ka Lam Tang:** Writing—review and editing (equal). **Helen Shuk Wah Ma:** Methodology (equal); writing—review and editing (equal). **Benjamin Hon Kei Yip:** Validation (equal); writing—review and editing (equal). **Daisy Dexing Zhang:** Writing—review and editing (equal). **Sarah Bowen:** Conceptualization (equal); methodology (equal); supervision (lead); writing—review and editing (equal). **Samuel Yeung Shan Wong:** Conceptualization (lead); funding acquisition (lead); methodology (equal); supervision (lead); writing—review and editing (equal).

## DECLARATION OF INTERESTS

None.

## CLINICAL TRIAL REGISTRATION DETAILS

This study was registered at ClinicalTrials.gov (Trial Registration Number: NCT04034732) and the Chinese Clinical Trial Register (Trial Registration Number: ChiCTR1900026247).

## Data Availability

The dataset generated and analyzed during the current study is available in the CUHK Research Data Repository.

## Appendix 1 Session summary of the 8‐week mindfulness‐based relapse prevention (MBRP).

**Table:**

Session	Core intentions	Mindful practice and cognitive exercise
(1) Automatic pilot and mindful awareness	Introduce the idea of ‘automatic pilot’ versus mindful awareness and explore how these are related to relapse Learn to bring awareness to the present moment using body sensations	Mindful practice: raisin exercise, body scan
(2) A new relationship with discomfort	Recognize triggers and experience them without reacting in a habitual way Observe thoughts, emotions, sensations and impulses when experiencing triggers and notice their transient nature Cultivate the sense of steadiness using the body and the mountain imagery	Mindful practice: body scan, urge surfing, mountain meditation Cognitive exercise: walking down the street
(3) From reacting to responding	Learn to return to the present moment using sensations of breathing Cultivate mindful awareness in daily life using 3‐step breathing space Identify needs underlying addictive behaviours	Mindful practice: awareness of hearing, breath meditation, 3‐step breathing space Cognitive exercise: false refuge
(4) Mindfulness in challenging situations	Cultivate a friendly awareness toward thoughts, emotions, body sensations and impulses experienced in a challenge situation Recognize one's individual relapse triggers Cultivate mindful awareness while walking	Mindful practice: awareness of seeing, sitting meditation (sound, breath, sensation, thought), 3‐step breathing space in a challenging situation, walking meditation Cognitive exercise: individual and common relapse risks
(5) Acceptance and skilful action	Explore what acceptance really means Cultivate acceptance toward unpleasant experiences, especially emotions and sensations Cultivate awareness in daily communication Cultivate mindfulness awareness during movement	Mindful practice: sitting meditation (sound, breath, sensation, thought, emotion), 3‐step breathing space (in pairs), mindful movement Cognitive exercise: discussion of acceptance and skilful action
(6) Seeing thoughts as thoughts	Notice thoughts as mental events Explore the role of thoughts in the relapse process	Mindful practice: sitting meditation (thoughts), 3‐step breathing space Cognitive exercise: thoughts and relapse, relapse cycle

*Note*: Adapted from Bowen S, Chawla N, Grow J, Marlatt GA. *Mindfulness‐based Relapse Prevention for Addictive Behaviors, Second Edition: A Clinician's Guide*. Guilford Publications; 2021 Feb 26.
